# How to Make a Paper Fan

**Published:** 1885-04

**Authors:** 


### HOW TO MAKE A PAPER FAN.

I recently required a dish to silver some paper on, and none could be obtained near where I live. I made a dish in the following manner : First cut out a block of wood the exact size and thickness of dish required. Then take a sheet of cartridge paper, paste it with flour paste and rub in the paste well, letting the paper be thoroughly soaked with it. Then place the paper evenly on the wooden block, turn down the edges smoothly and turn the corners back, rubbing them down well. Be veıy particular with the first sheet, because if you get that smooth, the rest is easy. Follow with another sheet of cartridge paper, turning the surplus or slack paper at the corners, the opposite direction to the last. Follow with five or six sheets of old newspaper in the same way, and cap with another sheet of cartridge. Put the block with the paper on it into an oven, and bake till dry. Then take out the block and trim the edges. Paint the outside of the paper dish with varnish. Pour some varnish inside the dish and let it soak in, and then pour off the surplus. Bake in the oven again. After the varnish is hard and dry, warm the dish until it is hot enough to melt paraffine waxr Pour some melted paraffine into it, and tilt it about till the bottom and sides are evenly rovered ; pour off the surplus, and when dry you can use for toning, developing, or even silvering paper. Of course the above is only recommended as a substitute for glass or porcelain when the latter cannot be readily obtained. Paraffine alone may be used if you like.—*Photo. Times.*

